# MODELING HETEROGENEITY IN COGNITIVE TRAJECTORIES IN THE FRAMINGHAM HEART STUDY

**DOI:** 10.1093/geroni/igad104.2952

**Published:** 2023-12-21

**Authors:** Yuan Fang, Jiachen Chen, Joanne Murabito, Kathryn Lunetta

**Affiliations:** Binghamton University, Binghamton, New York, United States; Boston University School of Public Health, Boston, Massachusetts, United States; Boston University Chobanian & Avedisian School of Medicine, Boston, Massachusetts, United States; Boston University School of Public Health, Boston, Massachusetts, United States

## Abstract

The prevalence of cognitive impairment in the population is growing. Maintaining good cognitive health is central to successful aging, independence, and well-being. Although older age is generally associated with worse cognitive function, there are substantial heterogeneities in the rate of cognitive decline across different cognitive domains and in terms of the clinical onset age of dementia. Recently, harmonized factor scores measuring global cognitive function for the memory, executive function, and language domains have been created in the Framingham Heart Study (FHS). In this work, we identified FHS participants with two or more repeated factor scores after the age of 60 (n=2339; 57% female; 17% APOE-ϵ4 carriers; 64% attended colleges) and were dementia-free at the baseline visits. We fitted latent class mixed models (LCMM) to cluster cognitive trajectories from all three domains using the harmonized factor scores. Non-linear trends with age in the trajectories were modeled via piecewise linear LCMM models followed by stepwise selections to select cluster-specific change points. We identified different latent classes of participants, some characterized by an early cognitive decline before age 70 as compared to late decliners, across different domains. Using 10-fold cross-validation, we also showed that the subgroupings of participants are stable. Our findings indicate class-related differential patterns in cognitive aging in the FHS. Future associations between the identified subclasses, cognitive outcomes, and physical or biological markers may advance the knowledge of multiple pathways of cognitive aging.

